# The role of personality, disability and physical activity in the development of medication-overuse headache: a prospective observational study

**DOI:** 10.1186/s10194-018-0863-1

**Published:** 2018-05-25

**Authors:** Louise S. Mose, Susanne S. Pedersen, Birgit Debrabant, Rigmor H. Jensen, Bibi Gram

**Affiliations:** 1 Department of Neurology, Hospital Southwest Jutland, Esbjerg, Denmark; 2 The Research Unit of Health Science, Hospital of Southwest Jutland, Esbjerg and Department of Regional Health Research, University of Southern Denmark, Odense, Denmark; 3 Department of Psychology, University of Southern Denmark, Odense, Denmark; 4 Department of Cardiology, Odense University Hospital, Odense, Denmark; 5 Epidemiology, Biostatistics and Biodemography Department of Public Health, University of Southern Denmark, Odense, Denmark; 6 Danish Headache Centre, Department of Neurology, Rigshospitalet-Glostrup, University of Copenhagen, Copenhagen, Denmark

**Keywords:** Migraine, Medication-overuse headache, Personality, Disability, Physical activity

## Abstract

**Background:**

Factors associated with development of medication-overuse headache (MOH) in migraine patients are not fully understood, but with respect to prevention, the ability to predict the onset of MOH is clinically important. The aims were to examine if personality characteristics, disability and physical activity level are associated with the onset of MOH in a group of migraine patients and explore to which extend these factors combined can predict the onset of MOH.

**Methods:**

The study was a single-center prospective observational study of migraine patients. At inclusion, all patients completed questionnaires evaluating 1) personality (NEO Five-Factor Inventory), 2) disability (Migraine Disability Assessment), and 3) physical activity level (Physical Activity Scale 2.1). Diagnostic codes from patients’ electronic health records confirmed if they had developed MOH during the study period of 20 months. Analyses of associations were performed and to identify which of the variables predict onset MOH, a multivariable least absolute shrinkage and selection operator (LASSO) logistic regression model was fitted to predict presence or absence of MOH.

**Results:**

Out of 131 participants, 12 % (*n*=16) developed MOH. Migraine disability score (OR=1.02, 95 % CI: 1.00 to 1.04), intensity of headache (OR=1.49, 95 % CI: 1.03 to 2.15) and headache frequency (OR=1.02, 95 % CI: 1.00 to 1.04) were associated with the onset of MOH adjusting for age and gender. To identify which of the variables predict onset MOH, we used a LASSO regression model, and evaluating the predictive performance of the LASSO-mode (containing the predictors MIDAS score, MIDAS-intensity and –frequency, neuroticism score, time with moderate physical activity, educational level, hours of sleep daily and number of contacts to the headache clinic) in terms of area under the curve (AUC) was weak (apparent AUC=0.62, 95% CI: 0.41-0.82).

**Conclusion:**

Disability, headache intensity and frequency were associated with the onset of MOH whereas personality and the level of physical activity were not. The multivariable LASSO model based on personality, disability and physical activity is applicable despite moderate study size, however it can be considered as a weak classifier for discriminating between absence and presence of MOH.

## Background

Migraine is a primary headache disorder characterized by recurring attacks, often described as a one-sided pulsating headache. The mean prevalence of current migraine in adults is around 15%, 8% in men versus 17.6 % in women in Europe [1]. Due to frequent pain-relieving medication intake, migraineurs are predisposed to develop medication-overuse headache (MOH) [2–5], which is defined as a chronic headache disorder developed as a consequence of regular overuse of acute or symptomatic headache medication [6]. Symptoms of MOH are often an aggravation and a chronification of the primary headache disorder [7]. Migraine is the primary headache for many MOH patients, however, not all migraine patients develop MOH. Clearly recognized predictors of developing MOH are unknown, but potential risk factors such as headache frequency, daily smoking, inactivity, comorbid pain conditions, anxiety and depression have all been discussed in literature [8]. Furthermore, also comorbidity with psychiatric conditions and psychological destress may negatively and significantly modify the outcome for migraine patients [9].

The psychological profile of migraine patients developing MOH is not fully understood, but the association between migraine and personality has been a topic of interest for many years [10, 11]. A common, well-established approach for describing personality traits is the Five Factor Model of personality [12, 13]. Theoretically, the Five Factor Model approaches personality as a hierarchical system of personality in terms of five basic independent domains: *Neuroticism, extroversion, openness to experience, agreeableness,* and *conscientiousness* [14]. Previous studies have demonstrated that a high score on the personality domain neuroticism is correlated with depression [13]. Both migraine (-MOH) and migraine patients (+MOH) have increased risk of developing depression, but migraine patients (+MOH) have a higher prevalence of depression compared to migraine patients (-MOH) [15]. Both the personality domain neuroticism and extraversion have been linked to general health [16] and a high score on neuroticism has been linked to migraine [17–19]. Additional research is needed to clarify the association between migraine and personality characteristics [10]. The Five Factor Model is commonly used to evaluate psychopathology, however, to the best of our knowledge only few studies have investigated personality characteristics of migraine patients using this framework [17, 20].

It is well-documented that headache patients in general experience decreased quality of life and disabilities [21, 22]. MOH patients frequently experience severe disability as compared to migraine patients without medication overuse [23, 24], but the role of disability in chronification is so far unknown. In the Global Burden of disease (GBD) study from 2016, migraine was estimated to be the main reason for years lived with disability in the age group 15-49, which represented a significant rise from the year before. The explanation was that MOH in GBD 2016 was attributed to the antecedent headache disorder instead of being reported separately [25, 26]. The 2016 GBD findings further underline the disabling role of migraine in general and in particular MOH as a sequela. Studies have shown that MOH patients have higher Migraine Disability Assessment Questionnaire scores (MIDAS) than migraine patients without medication overuse, indicating a higher degree of disability [21, 23, 24, 27]. Still, there is limited research on whether the MIDAS score in migraine patients correlates with the onset of MOH.

In a systematic review, Rhodes & Smith [28] concluded in correlation between personality characteristics and physical activity that neuroticism is negatively correlated with physical activity, while extraversion and conscientiousness have a positive correlation to physical activity [28]. A recent Danish cross-sectional study found an association between inactivity, daily smoking, obesity, and MOH [29]. Similarly, a population-based study investigating risk factors for a new onset of MOH in chronic headache patients found that physical inactivity and smoking were risk factors for developing MOH [8]. Evidence is lacking on the role of different intensity levels of physical activity in the development of MOH among migraine patients.

From a clinical point of view, knowledge of risk factors for the onset of MOH in patients with an established migraine diagnosis is paramount to prevent development of MOH. Factors such as personality, disability and physical activity level may be interesting as possible predictors of MOH, and at the same time, easy to establish and asses through the patients’ medical history. Therefore, the aims of this study were firstly to investigate if personality, disability and physical activity level of migraine patients are associated with the development of MOH in a group of migraine patients who are in active treatment and secondly, to analyze to which extend these factors together can predict the onset of MOH. We hypothesized that personality, disability and level of physical activity varied between migraine patients (+MOH) and migraine patients (-MOH).

## Methods

### Design and participants

The design was a single-center prospective observational study of patients in active treatment recruited from the multidisciplinary Danish Headache Clinic, Hospital of Southwest Jutland in Denmark, between October 2015 and June 2017. Patients were eligible to participate, if they were between 18-65 years of age and had a primary diagnosis of migraine according to the diagnostic criteria from ICHD-III beta [6]. Exclusion criteria were presence of MOH based on diagnostic criteria of chronic migraine and MOH from ICHD-III beta [6] at inclusion. To minimize any errors and ambiguity in the personality data, we excluded patients with severe comorbid untreated depression or anxiety and patients diagnosed with personality disorders. All patients received standard treatment at the Headache Clinic, which included consultations with a neurologists or headache nurse every three months. As standard, patients were informed about the risk and criteria for MOH at the initial consultation. In between consultations, patients had the opportunity to contact a headache nurse by phone or mail.

### Procedure

Patients were informed about the study by the neurologists at the consultations in the Headache Clinic. For logistic reasons, it was only possible to include patients two days a week and to extend the recruitment period to 20 months. Number of consultations in the clinic and years diagnosed with migraine varied, however all included patients were seen regularly in the clinic. Patients completed questionnaires regarding personality, extent of disabilities due to migraine, and physical activity level at the inclusion. Throughout the study period, patients’ were followed regularly in the Headache clinic and during these consultations patients’ self-reported headache diaries together with the physician’s examination and assessment formed the basis of a confirmed diagnosis. Information about patients who developed MOH were obtained from the hospital electronic health records in June 2017 [6]*.*

### Ethics, consent and permissions

This study was part of a larger study that was approved by the Regional Committees on Health Research Ethics for Southern Denmark (ID S-20140114). It was conducted according to the Helsinki Declaration, meaning all patients were informed both orally and in writing prior to giving written informed consent. Permission was obtained from the Danish Data Protection Agency (2008-58-0035).

## Measurements

### NEO Five-Factor Inventory

We assessed personality with the Danish version of NEO Five-Factor Inventory questionnaire (NEO-FFI-3) [30]. The questionnaire consists of 60 items and is a brief version of the original NEO-PI-R [31]. For pragmatic reasons, the short version was chosen as it is less burdensome to patients as compared to the original version with 240 items. The questionnaire is designed as a hierarchical measure with personality seen as five well-established domains, which is also referred to as the Five Factor Model of personality. Each domain is assessed by means of 12 questions. The five domains are: (i) *Neuroticism* (e.g. the tendency to experience negative emotions, such as anxiety, fear, and frustration); (ii) *Extraversion* (e.g. the tendency to be outgoing and talkative); (iii) *Openness to experience* (e.g. the tendency to be creative and imaginative); (iv) *Agreeableness* (e.g. the tendency to be empathic and altruistic); and (v) *Conscientiousness* (e.g. efficient, organized, and having self-control). All questions are answered on a five-point Likert scale from “totally disagree to “totally agree”. For each domain a t-score is calculated as the sum of the 12 items’ score ranging from 12-60 [30]. In the current study, the internal consistency of the domains ranged from 0.74-0.90, measured by Cronbach’s alpha, which is considered satisfactory.

### Migraine Disability Assessment

To quantify the extent of disability, the MIDAS questionnaire was used, which is one of the most frequently used measures to assess disability in migraine patients [32–34]. MIDAS consists of 5 items that captures information on disability on a four-point score. The grade of disability is scored as the sum of days with headache during the previous three months that prevented patients from or reduced productivity by at least 50% with respect to work/school, housework, and social/leisure activities. Furthermore, MIDAS consists of two additional questions on number of days with headache during the previous three months and intensity of headache measured on a numeric rating scale ranging from 0-10 where 0 is “no pain” and 10 is “worst imaginable pain”.

### Physical Activity Scale

To measure physical activity level, the questionnaire Physical Activity Scale 2.1 (PAS 2.1) was used [35]. In PAS 2.1, the patients were asked to specify number of hours and minutes in an average 24-hour day spent on physical activity categorized as i) sleeping, ii) work related sitting/standing/walking and heavy physical work, iii) transportation to or from work (walking/cycling to work), and iv) sedentary leisure time activities (e.g. TV-viewing). Additionally, PAS 2.1 provided estimates on hours and minutes on a weekly basis spent on physical activity at three different intensity levels: 'Light', 'moderate' and 'vigorous' physical activity.

## Statistical analyses

The outcome of interest in this study was whether patients developed MOH during the study period or not. Baseline demographic characteristics comparing the two groups migraine (+MOH) and migraine (-MOH) were calculated using the chi-square test for larger samples and Fisher’s exact for samples less than five categorical data and Mann-Whitney U test for data with skewed distribution and unpaired t-test for data following normal distribution. For hypothesis testing we used two-tailed test. *P*-values of < 0.05 were considered statistically significant.

### Associations between development of MOH, personality, disability and physical activity

Using logistic regression, we investigated whether MOH onset was associated with personality characteristics, disabilities or physical activity level. Each of the following variables were tested in a separate regression model: Unemployment, neuroticism, extraversion, openness, agreeableness, conscientiousness, MIDAS score, MIDAS-intensity and MIDAS-frequency, and physical activity level divided into hours weekly on light, moderate or vigorous activity. All regression models were adjusted for age and gender to avoid confounding effects on both personality score and development of MOH [4, 30, 36–38].

### Predicting presence or absence of MOH

To investigate the ability of our variables to jointly predict onset of MOH, we considered a multivariable prediction model obtained by least absolute shrinkage and selection operator (LASSO) regression. This penalized regression method allows for the integration of a large number of possible correlated predictors into one model and to select amongst these despite a small sample size. The following predictors were included: age, gender, civil status, educational level, primary diagnosis, contacts to the headache clinic, the five NEO-FFI-3 domains as separate variables, disability using MIDAS score, intensity and frequency, measurements from PAS 2.1 on times spent for sleeping, sedentary leisure time activities and times for light activities, moderate activities or vigorous activities and a binary variable indicating if patients were unemployed. Remaining variables from the PAS 2.1 assessing different activity levels during work times were not included, since they were not available for patients without employment. Educational level was included both as continuous and as categorical variable. We only used data from patients without missing data in any of the included covariates. Variable standardization prior to fitting was applied, but reported Odds ratios (OR) are returned on the original scale. Due to the LASSO penalty, ORs are biased towards one for the benefit of improved predictions. The tuning parameter controlling the strength of the penalty was chosen to maximize the goodness-of-fit in an 8-fold cross validation. Folds were chosen randomly, but each fold contained two (+MOH) patients. As goodness-of-fit-measure we used the area under the ROC curve (AUC). After the tuning parameter had been determined, we calculated the AUC in the complete dataset together with its 95% confidence interval. Because the AUC value of our prediction model for new samples from the same population is expected to be below the calculated apparent AUC (especially because of the previous model selection incorporated in the LASSO approach), we applied bootstrap resampling as described in Steyerberg [39] to calculate an optimism-corrected AUC-value. We used 500 bootstrap samples, but discarded those for which the LASSO logistic regression failed to converge. At the same time and using the same approach, we calculated bootstrap based corrections for the lower and upper bound of the corresponding confidence interval.

Statistical analyses were performed with StatalC14 (StataCorp LP, College Station, Texas). We used the statistics software R (version 3.3.2) together with the packages glmnet version 2.0-10 [40], ROCR version 1.0-7 [41], pROC version 1.10.0 [42] and caret version 6.0-76 [43] to carry out the LASSO logistic regression model, calculate the AUC and its confidence intervals and to plot the ROC curve.

## Results

A total of 156 patients were informed about the study and 131 accepted to participate. Two patients did not want to participate, as they felt uncomfortable about answering the questionnaires, while 23 patients failed to return the questionnaires. Of the 131 included patients, 119 (91%) received prophylactic treatment for migraine at inclusion and patients had a mean [range] follow-up time of 361[18-631] days. There were no statistically significant differences between non-responders and included patients regarding age, gender and primary headache diagnoses (all *p*-values > 0.05).

### Clinical characteristics

Sixteen migraine patients (12%) developed MOH, while 88% (*n*=115) were still migraine patients without MOH at the end of the study period. The majority of migraine patients (-MOH) were women 87% (*n*=100) with a mean (SD) age of 39.2 (13) years. The distributions of primary headache diagnosis in the migraine (-MOH) group were 38 % (*n*=44) had tension-type headache (TTH) as comorbidity, while 62% (*n*=71) had only migraine. Also in the migraine (+ MOH) group, women were predominant by 94 % (*n*=15) with a mean (SD) age of 37.3(13). In this group migraine was primary headache diagnosis for 50% (*n*=8) while 50 % (*n*=8) had comorbidity migraine and TTH. The migraine (+MOH) group had a significantly higher numbers of contacts to the Headache Clinic during the study period; median (IQR) contacts 8.5 (4 to 10) as compared to the migraine (-MOH) group with median (IQR) contacts 6 (3 to 7) (*p*= 0.028). No other statistically significant differences in demographic and headache characteristics were observed between the groups. Characteristics are summarized in Table 1.

**Table 1:** Patients’ demographic and headache characteristics

	Migraine (-MOH) (*n* = 115)	Migraine (+ MOH) (*n* = 16)	All participants (*n* = 131)	*P*- value
Age (years)(mean±SD)	39.2±13	37.3±13	39.0±13	NS
Sex n (%)				
Female	100 (87)	15 (94)	115 (88)	NS
Male	15 (13)	1 (6)	16 (12)	
Civil status n (%)				
Single	25 (22)	3 (19)	28 (21)	NS
Cohabiting	90 (78)	13 (81)	103 (79)	
Educational level n (%)				
Primary/secondary school	16 (14)	5 (31)	21 (16)	NS
Vocational/High school	55 (48)	7 (44)	62 (47)	
Bachelor or higher degree	44 (38)	4 (25)	48 (37)	
Working status n (%)				
Employed/student	91 (79)	13 (81)	104 (82)	NS
Unemployed/sickness benefits/social welfare	24 (21)	3 (19)	27 (18)	
Sleep (hours daily) (mean±SD)	8±1	8±1	8±1	NS
Primary diagnosis n (%)				
Migraine	71 (62)	8 (50)	79 (60)	NS
Migraine + Tension Type Headache	44 (38)	8 (50)	52 (40)	
Contacts headache clinic median [IQR]	6 [3-7]	8.5 [4-10]	5 [3-8]	0.028

Differences between groups on normal distributed data were tested using unpaired t-test and Chi-square test for samples >5 and Fisher’s exact for samples < 5. Data are presented as mean ± standard deviation (SD) or as numbers with percentages (%) in brackets. For skewed data Wilcoxon Mann-Whitney test were used and data were presented as median and interquartile range [IQR] in brackets. *P*-values < 0.05 were considered as statistically significant for all tests.

Comparison of personality characteristics between groups showed no statistical differences. Migraine (+MOH) had significantly higher headache intensity median (IQR) 7(6.5-8) as compared to the migraine (-MOH) group with median (IQR) of 6 (5-7), (*p*=0.041). Headache frequency for the previous three months, were also significantly higher among the migraine (+MOH) group with median (IQR) of 46 (28.5-87.5), compared to migraine (-MOH) with median (IQR) of 30 (15-54), (*p*=0.017) (Table 2). Overall, neither the migraineurs (-MOH) nor the migraineurs (+MOH) were physically active as they spent only few hours weekly on light physical activity, even fewer hours at moderate physical activity and almost no time on vigorous physical activity. No statistical significant differences were found between the groups regarding level of physical activity (Table 2).

**Table 2:** Comparison of personality, disability and physical activity levels between migraine (-MOH) and migraine (+MOH). All data are presented as medians [interquartile ranges]

	Migraine (-MOH) (*n*= 115)	Migraine (+MOH) (*n*= 16)	All participants (*n*=131)	*P*-value
Neuroticism (12-60)	35 [28-42]	38.5 [31- 46.5]	35 [28-42]	NS
Extraversion (12-60)	39 [34-44]	39 [30.5-41]	39 [33-44]	NS
Openness (12-60)	37 [33-42]	34.5 [34-43]	37 [33-42]	NS
Agreeableness (12-60)	44 [39- 48]	44.5 [39- 48.5]	44 [39- 48]	NS
Conscientiousness (12-60)	47 [43-51]	46.5 [39.5- 50]	47 [42-51]	NS
MIDAS score (0-270)	32 [16- 57]	42 [28- 94.5]	33 [17-58]	NS
MIDAS-frequency (days last 3 months)	30 [15-54]	46 [28.5-87.5]	30 [18- 63]	0.017
MIDAS-intensity (NRS 0-10)	6 [5-7]	7 [6.5-8]	7 [5-7]	0.041
Light physical activity (hours/week)	5.25 [3-10]	7 [4-13]	6 [3-10]	NS
Moderate physical activity(hours/week)	2.5 [1-5]	2 [0.5-4]	2.5 [0.5-5]	NS
Vigorous physical activity (hours/ week)	0 [0-2]	0 [0-1.5]	0 [0-2]	NS

Differences between groups were tested using Wilcoxon Mann-Whitney test. *P*-values< 0.05 were considered as statistically significant for all tests. NEO-FFI-3: NEO Five-Factor Inventory. MIDAS: Migraine disability assessment questionnaire. PAS 2.1: Physical Activity Scale questionnaire

### Associations between development of MOH, personality, disability and physical activity

When adjusting for age and gender in the logistic regressions analysis for the onset of MOH, no significant differences in odds were found with respect to unemployment (OR=0.967, 95% CI: 0.24 to 3.77). The personality domains neuroticism (OR=1.06, 95 % CI: 0.99 to 1.13), extraversion (OR=0.96, 95 % CI: 0.88 to 1.04), openness (OR=0.99, 95 % CI: 0.91 to 1.08), agreeableness (OR=1.00, 95 % CI: 0.93 to 1.08) and conscientiousness (OR=0.95, 95 % CI: 0.87 to 1.03) were not associated with onset of MOH in migraine patients.

Analyses on the relationship between MIDAS score and MOH demonstrated significant association between MIDAS score (OR=1.02, 95 % CI: 1.00 to 1.04) and intensity of headache (OR=1.49, 95 % CI: 1.03 to 2.15) and between MIIDAS score and headache frequency (OR=1.02, 95 % CI: 1.00 to 1.04).

The three variables describing levels of physical activity were not significantly associated with onset of MOH; light activity (OR=1.00, 95 % CI: 0.93 to 1.08), moderate activity (OR=0.87, 95 % CI: 0.70 to 1.07) and vigorous activity (OR=0.91, 95 % CI: 0.66 to 1.26). All association analyses using logistic regression are summarized in Table 3.

**Table 3:** Associations between development of MOH and personality, disability and physical activity level

Covariates	OR	95% CI	*P*-value
Unemployment (*n*=131)	0.97	0.24-3.77	0.961
Light physical activity (hours/week)(*n*=130)	1.00	0.93-1.08	0.947
Moderate physical activity (hours/week)(*n*=130)	0.87	0.70-1.07	0.193
Vigorous physical activity (hours/week)(*n*=131)	0.91	0.66-1.26	0.588
MIDAS score (0-270)(*n*=131)	1.02	1.00-1.04	0.032
MIDAS-intensity (NRS 0-10) (*n*=131)	1.49	1.03-2.15	0.034
MIDAS-frequency (days last 3 months) (*n*=131)	1.02	1.00-1.04	0.032
Neuroticism score (12-60) (*n*=131)	1.06	0.99-1.13	0.069
Extraversion score (12-60) (*n*=131)	0.96	0.88-1.04	0.275
Openness score (12-60) (*n*=131)	0.99	0.91-1.08	0.763
Agreeableness score (12-60) (*n*=131)	1.00	0.93-1.08	0.970
Conscientiousness score (12-60) (*n*=131)	0.95	0.87-1.03	0.206

Values are adjusted odds ratio and their 95 % CI and *P*-values from multivariable regression model with age and gender as covariates. The results are obtained from 12 different regressions

### Predicting presence or absence of MOH

Two patients out of 131 were excluded from this analysis due to missing values. Table 4 illustrates our multivariable prediction model and shows the covariates selected by the multivariable LASSO logistic regression together with the estimated ORs.

**Table 4:** Variables selected by the LASSO logistic regression

Covariates	OR (LASSO)	OR	95% CI (OR)
Sleep (hours daily)(*n*=129)	1.032	1.214	0.831-1.812
Moderate physical activity (hours/week) (*n*=129)	0.983	0.906	0.710-1.077
MIDAS score (*n*=129)	1.002	1.002	0.990-1.014
MIDAS-intensity (NRS 0-10) (*n*=129)	1.006	1.011	0.987-1.034
MIDAS-frequency (days last 3 months) (*n*=129)	1.210	1.564	1.042-2.517
Neuroticism score (12-60) (*n*=129)	1.017	1.052	0.981-1.133
Educational level (*n*=129)	0.794	0.586	0.233-1.396
Contacts headache clinic (*n*=129)	1.010	1.068	0.916-1.237

The table shows estimated (shrunken) odds ratio for the selected variables. These are complemented by odds ratios and their 95% CI afterwards obtained from an ordinary multivariable logistic regression model using the same variables. Odds ratios correspond to the variables’ original scale

The predicted odds for developing MOH increased by 21% (OR=1.210) by each unit of headache frequency reported. The predicted odds increased by 0.2% (OR=1.002) for each additional MIDAS score point, and 0.6% (OR=0.006) for each unit of intensity of headache reported.

The predicted odds for experiencing MOH estimated by the LASSO model were 3.2% higher (OR=1.032) for each additional hour of sleep, and decreased by 1.7% (OR=0.983) for each hour spent on moderate physical activity. Regarding personality domains, an additional unit in the neuroticism score increased the odds by 1.7% (OR=1.017). Each additional level of education decreased the odds by 20.6% (OR=0.794) and each additional contact to the Headache clinic increased the odds by 1% (OR=1.010). Remaining covariates were not part of the selected model.

**Fig. 1:**
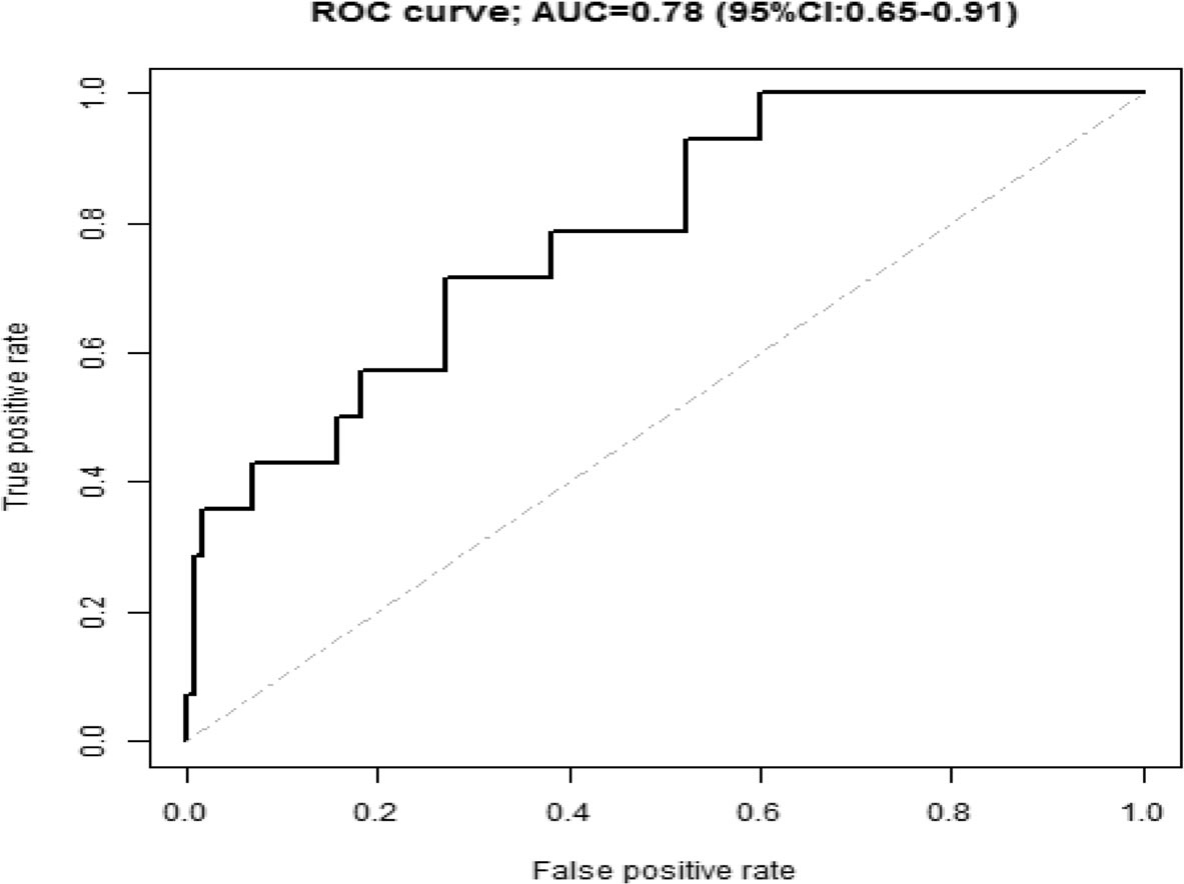
ROC curve and area under the curve (AUC) for the prediction model obtained by the LASSO logistic regression. Included predictors were: MIDAS score, MIDAS-intensity and –frequency, neuroticism score, time with moderate physical activity, education level, hours of sleep daily and contacts headache clinic

Evaluating the predictive performance of the LASSO-model, we obtained the ROC curve, shown in Fig. 1 together with an apparent area under the ROC curve (AUC) of 0.78 (95% CI: 0.65-0.91) in our sample. By obtaining the ROC curve we assess the ability of the predictors in the model to discriminate between absence and presence of MOH. The curve is obtained by the score divided from the LASSO logistic regression and based on the included predictors.

As the sample had already been used for model selection, this estimate of model performance is overly optimistic. During the following bootstrap procedure, 141 of the 500 bootstrap samples were discarded due to convergence problems during the estimation procedure. Using the remaining bootstrap samples, we obtained an optimism estimate of 0.16 resulting in a corrected AUC of 0.62. Similarly, the corrected 95% CI for the AUC was 0.41-0.82. The AUC presents a measurement of discrimination, that is, the ability of the model to correctly classify the onset of MOH. Given the fact that the corrected AUC is 0.62 (95% CI: 0.41-0.82), our model (containing the predictors MIDAS score, MIDAS-intensity and –frequency, neuroticism score, time with moderate physical activity, educational level, hours of sleep daily and number of contacts to the headache clinic)) the model can be considered as an weak classifier for discriminating between absence and presence of MOH.

## Discussion

The main findings of the present study were that the logistic regressions indicated that the headache intensity and headache frequency were associated with onset of MOH and therefore could be factors to take into account to prevent the development of MOH. This could have important implications for clinicians and highlights that the intensity and frequency of headache may help identify the sub group at risk of developing MOH. For this study, MIDAS questionnaire was chosen as an instrument, however other instruments measuring intensity and frequency (i.e. headache diary) could have been applicable. Furthermore, when comparing migraine patients (-MOH) and migraine patients (+MOH), patients developing MOH reported higher intensity and frequency of headache using the MIDAS questionnaire as compared to the rest of the included migraine patients. This finding is consistent with a review that showed high headache frequency to be an important modifiable risk factor in migraine chronification progression [44] and with previous studies stating that headache frequency in particular may be a risk factor for onset MOH [8, 45]. Martelletti shows that MOH must be considered as sequela of chronic migraine and in light of that, it is beneficial to focus on how to reduce headache frequency among migraine patients to avoid MOH as a consequence [46].

Bigal et al. [47] investigated psychological profiles including the role of personality characteristics in headache chronification and observed that episodic headache patients undergoing chronification had a different personality profile compared to patients with episodic headaches [47]. In contrast, when we compared personality characteristics between migraine (-MOH) and migraine (+MOH) patients, we were unable to detect any differences between the groups. This can be caused by the fact that all patients at starting point were migraine patients without MOH, and the two groups therefore remain very similar in personality characteristics, unaffected by the chronification process related to developing MOH. Generally, few studies have investigated associations between different headache types and personality, and the results have been ambiguous [48]. Furthermore, different measurements of personality make studies difficult to compare. For this study we chose the NEO-FFI-3 which describes personality as five domains with 12 items each without the underlying in-depth facet score [31]. This facet scores could potentially increase the sensitivity of the personality evaluations, making it possible to detect subtle differences that our method could not. This could explain the insignificant differences between (+MOH) and (-MOH) in the current study.

MOH patients tend to be more physically inactive as compared to migraine patients [8]. According to Westergaard et al. the association between MOH and inactivity might be due to the fact that MOH patients have developed an inactive lifestyle in order to avoid triggering migraine attacks [29]. In this study, both patients with migraine (-MOH) and migraine (+MOH) spent very few hours on physical activities per week, which probably could be caused by headache burden. The difficulty of performing physical activity among migraine patients in association to development of MOH seems to be irrelevant as the odds for MOH only decreased by 1.7% (OR=0.983) for each hour spent on moderate physical activity. However, a study on physical activity and migraine treatment found that regular physical activity has beneficial effects on headache intensity and frequency, duration of headache attacks and patients well-being [49]. When MOH patients are physically inactive there could be a risk of maintaining MOH in an inappropriate circular process with inactivity, worsening in headache and increased medical intake.

This study is the first to investigate the predictive performance of models based on personality, disability and physical activity in predicting onset of MOH. It is challenging, but clinically relevant, to identify patients at risk of developing MOH and therefore studies developing predictive models of headache chronification are important [50]. In our prediction model we could not determine a strong causality between the included factors of personality, disability and physical activity level in the onset of MOH, however, we are not able to reject it either. Our findings substantiates that multiple factors potentially contributes to the onset of MOH, which is in line with another similar predictive study [20] were they found an AUC of 0.76 when including factors of personality, gene polymorphisms, headache characteristics and lifestyle. In both studies the AUC showed a weak model classifier for the onset of MOH.

As strength in this study the questionnaires used were not time consuming, which must be considered as an advantage, because it is easy to adopt in clinical practice. As a limitation we must consider the inclusion process since we only included patients two times per week. Therefore we are fully aware that this study represents only a sample of a larger migraine patient flow in the clinic. However this study indicates how many migraine patients developed MOH during treatment at a headache clinic. Further we are limited by investigating patients who were seen and treated regularly and therefore, we are unable to assess how many patients would have developed MOH if they had no treatment options, and if this could have caused other predictive factors to emerge. Migraineurs with particularly high medication intake are at increased risk of developing MOH [51]. However, detailed knowledge about prophylactic and acute medical treatment is not part of the current study, since the patients’ medical ordinations changed throughout the study. Overall, only 12% of the patients developed MOH during the study period and the small number of patients developing MOH is a limitation for immediate generalization of the results. This limits the power of the study and increases the risk for errors in both the estimated size as well as direction of effects. However, one way to handle these limitations is by looking at Gelman & Calin [52] who recommend a design calculation that provides a perspective on erroneous findings in small studies. The design calculation estimates the type S error, meaning the probability of an estimate being in the wrong direction, and type M errors being by which factor the magnitude of an effect is overestimated. Even though the dataset was small, we succeeded on characterizing the patients developing MOH and modeling our data via solid statistical methods highlighting important methods for assessing easy accessible clinical data in the prevention of MOH. The predictors included in the presented prediction model were those which best predicted presence or absence of MOH. Due to the small effective sample size of 16 cases of MOH in the dataset, the complexity of resulting models was a priori limited as larger models tend to be more prone to overfitting. Further, for penalized regression methods such as LASSO logistic regression, the number of selected predictors depends crucially on the chosen strength of the penalty. In this study, its choice was governed by cross-validation. Taking the variability incorporated in this procedure into account, a wider range of other prediction models including more or less predictors becomes plausible, see also Pfeiffer & Raymond [53] who estimated the rate of falsely included/excluded variables when applying LASSO logistic regression to a simulated dataset.

## Conclusion

This study showed that the intensity and frequency of headache were associated with MOH onset, while there were no associations between personality and physical activity level and MOH onset, respectively. Our findings support that focus on headache frequency and intensity is essential for targeting a subgroup of migraine patients at risk of developing MOH.

The identification of predictors of MOH may have important clinical implications - specifically in relation to early detection of patients at risk of developing MOH and in the documentation of appropriate instruments for this detection.

## Abbreviations

AUC: Area under the ROC curve is a measurement of discrimination, that is, the ability of the model to correctly classify the onset of medication-overuse headache
CI: Confidence Interval
LASSO: Least absolute shrinkage and selection operator
MIDAS: Migraine Disability Assessment Questionnaire
MOH: Medication-overuse headache defined according to the diagnostic criteria from ICHD-III beta
NEO-FFI-3: NEO Five-Factor Inventory questionnaire
NEO-PI-R: The revised NEO Personality Inventory is the longer version of NEO-FFI-3 containing 240 items.
OR: Odds Ratio
PAS 2.1: The Physical Activity Scale, which is a questionnaire.
ROC Curve: Receiver operating characteristic curve
TTH: Tension-type headache
